# New Minerals from Inclusions in Corundum Xenocrysts from Mt. Carmel, Israel: Magnéliite, Ziroite, Sassite, Mizraite-(Ce) and Yeite

**DOI:** 10.3390/ma16247578

**Published:** 2023-12-09

**Authors:** Chi Ma, Fernando Cámara, Luca Bindi, Vered Toledo, William L. Griffin

**Affiliations:** 1 Division of Geological and Planetary Sciences, California Institute of Technology, Pasadena, CA 91125, USA; 2 Dipartimento di Scienze della Terra “A. Desio”, Università degli Studi di Milano, Via Mangiagalli 34, I-20133 Milan, Italy; fernando.camara@unimi.it; 3 Dipartimento di Scienze della Terra, Università degli Studi di Firenze, Via La Pira 4, I-50121 Florence, Italy; luca.bindi@unifi.it; 4 Shefa Gems (A.T.M.) Ltd., Netanya 4210602, Israel; veredshefa@gmail.com; 5 ARC Centre of Excellence for Core to Crust Fluid Systems and GEMOC, Earth and Environmental Sciences, Macquarie University, Macquarie Park, NSW 2109, Australia

**Keywords:** magnéliite, Ti^3+^_2_Ti^4+^_2_O_7_, ziroite, ZrO_2_, sassite, Ti^3+^_2_Ti^4+^O_5_, mizraite-(Ce), Ce(Al_11_Mg)O_19_, yeite, TiSi, new minerals, corundum, Mt. Carmel, Israel

## Abstract

Our nanomineralogical investigation of melt inclusions in corundum xenocrysts from the Mt. Carmel area, Israel has revealed seven IMA-approved new minerals since 2021. We report here four new oxide minerals and one new alloy mineral. Magnéliite (Ti^3+^_2_Ti^4+^_2_O_7_; IMA 2021-111) occurs as subhedral crystals, ~4 μm in size, with alabandite, zirconolite, Ti,Al,Zr-oxide, and hibonite in corundum Grain 767-1. Magnéliite has an empirical formula (Ti^3+^_1.66_Al_0.13_Ti^4+^_0.15_Mg_0.10_Ca_0.01_Sc_0.01_)_Σ2.06_ (Ti^4+^_1.93_Zr_0.08_)_Σ2.01_O_7_ and the triclinic *P*$\bar{1}$ Ti_4_O_7_-type structure with the cell parameters: *a* = 5.60(1) Å, *b* = 7.13(1) Å, *c* = 12.47(1) Å, *α* = 95.1(1)°, *β* = 95.2(1)°, *γ* = 108.7(1)°, *V* = 466(2) Å^3^, *Z* = 4. Ziroite (ZrO_2_; IMA 2022-013) occurs as irregular crystals, ~1–4 μm in size, with baddeleyite, hibonite, and Ti,Al,Zr-oxide in corundum Grain 479-1a. Ziroite has an empirical formula (Zr_0.72_Ti^4+^_0.26_Mg_0.02_Al_0.02_Hf_0.01_)_Σ1.03_O_2_ and the tetragonal *P*4_2_/*nmc* zirconia(HT)-type structure with the cell parameters: *a* = 3.60(1) Å, *c* = 5.18(1) Å, *V* = 67.1(3) Å^3^, *Z* = 2. Sassite (Ti^3+^_2_Ti^4+^O_5_; IMA 2022-014) occurs as subhedral-euhedral crystals, ~4–16 μm in size, with Ti,Al,Zr-oxide, mullite, osbornite, baddeleyite, alabandite, and glass in corundum Grain 1125C1. Sassite has an empirical formula (Ti^3+^_1.35_Al_0.49_Ti^4+^_0.08_Mg_0.07_)_Σ1.99_(Ti^4+^_0.93_Zr_0.06_Si_0.01_)_Σ1.00_O_5_ and the orthorhombic *Cmcm* pseudobrookite-type structure with the cell parameters: *a* = 3.80(1) Å, *b* = 9.85(1) Å, *c* = 9.99(1) Å, *V* = 374(1) Å^3^, *Z* = 4. Mizraite-(Ce) (Ce(Al_11_Mg)O_19_; IMA 2022-027) occurs as euhedral crystals, <1–14 μm in size, with Ce-silicate, Ti-sulfide, Ti,Al,Zr-oxide, ziroite, and thorianite in corundum Grain 198-8. Mizraite-(Ce) has an empirical formula (Ce_0.76_Ca_0.10_La_0.07_Nd_0.01_)_Σ0.94_(Al_10.43_Mg_0.84_Ti^3+^_0.60_Si_0.09_Zr_0.04_)_Σ12.00_O_19_ and the hexagonal *P*6_3_/*mmc* magnetoplumbite-type structure with the cell parameters: *a* = 5.61(1) Å, *c* = 22.29(1) Å, *V* = 608(2) Å^3^, *Z* = 2. Yeite (TiSi; IMA 2022-079) occurs as irregular-subhedral crystals, 1.2–3.5 μm in size, along with wenjiite (Ti_5_Si_3_) and zhiqinite (TiSi_2_) in Ti-Si alloy inclusions in corundum Grain 198c. Yeite has an empirical formula (Ti_0.995_Mn_0.003_V_0.001_Cr_0.001_)(Si_0.996_P_0.004_) and the orthorhombic *Pnma* FeB-type structure with the cell parameters: *a* = 6.55(1) Å, *b* = 3.64(1) Å, *c* = 4.99(1) Å, *V* = 119.0(4) Å^3^, *Z* = 4. The five minerals are high-temperature oxide or alloy phases, formed in melt pockets in corundum xenocrysts derived from the upper mantle beneath Mt. Carmel.

## 1. Introduction

Worldwide, super-reduced mineral assemblages are commonly associated with explosive volcanic events such as kimberlites, alkali basalts, and tholeiitic basalts, as well as in ophiolites linked to deep subduction along continental plate margins [1,2,3]. The origins of these assemblages have sparked debate, with some attributing them to human activities [4]. However, the extensively documented xenoliths and xenocrysts discovered in small Cretaceous volcanoes and Plio-Pleistocene gem placer deposits at Mt. Carmel, Israel, play a crucial role in this discussion. The geological context, along with thorough geochemical analysis and precise geochronological data, effectively refute any plausible notion of human interference [3,5,6]. Many super-reduced minerals are identified as inclusions within xenoliths composed of corundum aggregates. The relationships between these different phases within melt inclusions have been crucial in interpreting the genesis of super-reduced magma-fluid systems.

A nanomineralogical investigation of melt inclusions found in corundum xenocrysts coming from volcanic centers and associated alluvial deposits in the Mt. Carmel area, Israel, has allowed us to discover seven IMA-approved new minerals since 2021: griffinite (Al_2_TiO_5_), magnéliite (Ti^3+^_2_Ti^4+^_2_O_7_), ziroite (ZrO_2_), sassite (Ti^3+^_2_Ti^4+^O_5_), mizraite-(Ce) (Ce(Al_11_Mg)O_19_), toledoite (TiFeSi), and yeite (TiSi) [7,8,9,10,11,12,13]. All of them have been approved by the IMA-CNMNC (Commission on New Minerals, Nomenclature and Classification) as requested before naming and reporting the finding of a new mineral phase. Griffinite has been already published in detail [14]. Toledoite will be published in a separate paper. Reported here are another five new minerals, adding more information on the origin of reduced high-temperature minerals from the upper mantle.

Magnéliite (IMA 2021-111), Ti^3+^_2_Ti^4+^_2_O_7_ (simply Ti_4_O_7_), is a new Ti-oxide mineral that corresponds to the first member of the homologous series of Ti-oxides (with Ti_n_O_2n−1_), known also as Magnéli phases [15]. The name is in honor of Arne Magnéli (1914–1996), for his pioneering work on the structural chemistry of transition-metal oxides.

Ziroite (IMA 2022-013), ZrO_2_, is a new Zr-oxide mineral with the *P*4_2_/*nmc* zirconia(HT)-type structure. The name is derived from its composition.

Sassite (IMA 2022-014), Ti^3+^_2_Ti^4+^O_5_ (simply Ti_3_O_5_), is another new Ti-oxide mineral with the *Cmcm* pseudobrookite-type structure. The name is in honor of Eytan Sass (b. 1932), a geologist at the Freddy and Nadine Herrmann Institute of Earth Sciences, Hebrew University of Jerusalem. He performed the excellent mapping work on Mt. Carmel that identified the various volcanic centers.

Mizraite-(Ce) (IMA 2022-027), Ce(Al_11_Mg)O_19_, is a new Ce-rich oxide mineral belonging to the magnetoplumbite-group [16], with the *P*6_3_/*mmc* magnetoplumbite-type structure. The name is after the Mizra river in the Mt. Carmel region, where some corundum xenocrysts investigated in this study (including Grain 198-8) come from alluvial deposits. The tributary Mizra river flows into the Kishon River.

Yeite (IMA 2022-079) is a new alloy mineral, TiSi, with the *Pnma* FeB-type structure. The name is in honor of Danian Ye (b. 1939), a mineralogist at the Institute of Geology and Geophysics, Chinese Academy of Sciences, for his many contributions to mineralogy and crystal chemistry.

## 2. Materials and Methods

The corundum xenoliths hosting the new minerals as inclusions occur in the pyroclastic ejecta from small Cretaceous basaltic volcanoes on Mt. Carmel and from placer gemstone deposits found in the terraces of the Paleocene to Pleistocene proto-Kishon river; the modern Kishon River drains Mt. Carmel and the tributary Mizra river and enters the sea near Haifa in northern Israel [2]. Much of the xenolith material in the paleoterrace deposits probably also is derived from Miocene and Pliocene basalt outcroppings in the drainage area of the Kishon River. The xenoliths occur as aggregates of skeletal corundum crystals that enclose melt pockets containing reduced mineral assemblages [1,2,17,18].

All the type materials are deposited in the mineralogy collection of the Università degli Studi di Milano, Via Mangiagalli, 34-20133 Milano, Italy. 

The type magnéliite in corundum Grain 767-1 from Mt. Carmel mount Corundum-SY is under the registration number MCMGPG-H2022-001.

The type ziroite in corundum Grain 479-1a from Mt. Carmel mount Corundum-18-1 is under the registration number MCMGPG-H2021-003.

The type sassite in corundum Grain 1125C1 from Mt. Carmel mount Corundum-18-1 is under the registration number MCMGPG-H2021-004.

The type mizraite-(Ce) in corundum Grain 198-8 from Mt. Carmel mount Corundum-18-1 is under the registration number MCMGPG-H2022-005.

The type yeite in corundum Grain 198c from Mt. Carmel mount Corundum-18-1 is under the registration number MCMGPG-H2021-002. This corundum grain also hosts the type griffinite (Al_2_TiO_5_; IMA 2021-110; [14]).

In order to characterize the composition and structure of the new minerals and associated phases, we used an electron probe microanalyzer (EPMA) and a high-resolution scanning electron microscope (SEM) with an X-ray energy dispersive spectrometer (EDS) and electron backscatter diffraction (EBSD). A ZEISS 1550VP Field-Emission SEM (ZEISS Group, Oberkochen, Germany) with an Oxford X-Max EDS was used for backscatter electron (BSE) imaging and fast elemental analysis. Quantitative WDS elemental microanalyses of the new minerals were carried out using a JEOL 8200 EPMA (JEOL Ltd., Tokyo, Japan) (15 kV and 10 nA, focused beam) and processed with the CITZAF correction procedure [19]. The focused electron beam is ~150 nm in diameter.

EBSD analyses at a submicrometer scale were performed using methods described by [20,21] for studies of micron-sized new minerals. An HKL EBSD system on the ZEISS 1550VP Field-Emission SEM was operated at 20 kV and 6 nA in focused beam mode with a 70° tilted stage and in a variable pressure mode (25 Pa). The EBSD system was calibrated using a single-crystal silicon standard. Experimental EBSD patterns allowed the collection of structural information and cell constants that were derived by matching with those of the structures of synthetic phases from the ICSD (Inorganic Crystal Structure Database).

Due to the small size of the samples, most of the physical properties (optical, hardness, fracture, cleavage, habit, density, etc.) were impossible to obtain.

## 3. Results

### 3.1. Magnéliite

**Figure 1:**
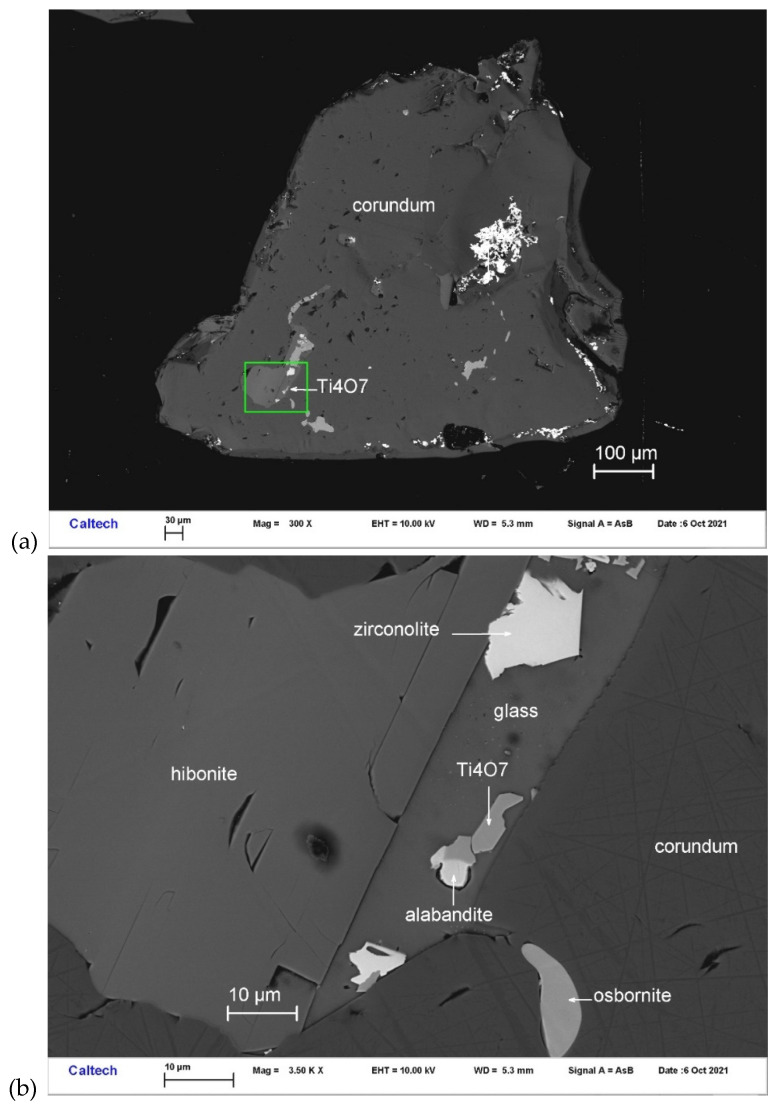
BSE images showing magnéliite (Ti_4_O_7_) in corundum Grain 767-1. The rectangular area in (**a**) is enlarged in (**b**).

Magnéliite occurs with alabandite, zirconolite, Ti,Al,Zr-oxide, and hibonite in one inclusion from corundum Grain 767-1 (Figure 1). Other inclusions in this corundum grain contain hibonite and osbornite. It is transparent, occurring as subhedral crystals ~4 μm in size. The Gladstone–Dale relationship [22] gives n = 2.423, obtained from the chemical composition and calculated density.

The chemical composition of magnéliite using EPMA (Table 1) shows an empirical formula (based on 7 O *pfu*) of (Ti^3+^_1.66_Al_0.13_Ti^4+^_0.15_Mg_0.10_Ca_0.01_Sc_0.01_)_Σ2.06_(Ti^4+^_1.93_Zr_0.08_)_Σ2.01_O_7_. The simplified formula is (Ti^3+^,Al)_2_Ti^4+^_2_O_7_. The ideal formula is Ti^3+^_2_Ti^4+^_2_O_7_, which requires Ti_2_O_3_ 47.36, TiO_2_ 52.64, total 100 wt%.

**Figure 2:**
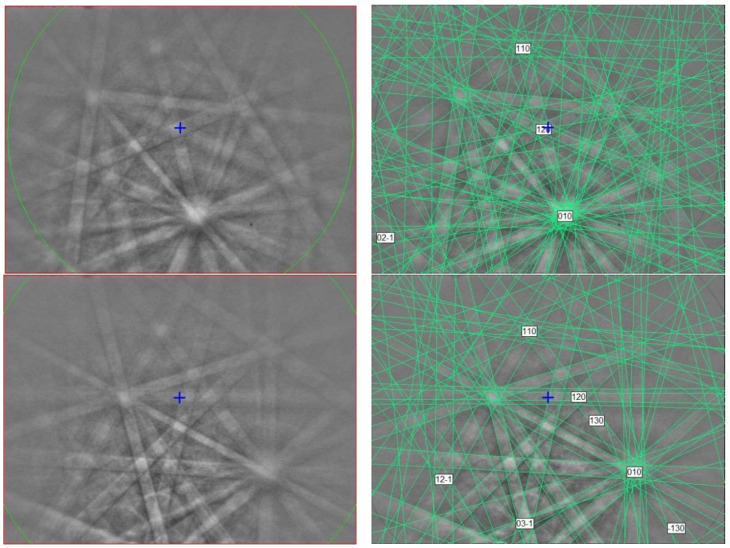
(**left**) EBSD patterns of the magnéliite crystal in Figure 1 at different orientations, and (**right**) the patterns indexed with the *P*$\bar{1}$ Ti_4_O_7_-type structure. Blue cross marks the pattern center.

The EBSD patterns can be indexed only by the *P*$\bar{1}$ Ti_4_O_7_-type structure and match the synthetic Ti_4_O_7_ cell from [23] (Figure 2), with a mean angular deviation of 0.32°–0.35°, revealing the following cell parameters: *a* = 5.60(1) Å, *b* = 7.13(1) Å, *c* = 12.47(1) Å, *α* = 95.1(1)°, *β* = 95.2(1)°, *γ* = 108.7(1)°, *V* = 466(2) Å^3^, and *Z* = 4. The calculated density is 4.30 g·cm^−3^ using the empirical formula and the unit-cell volume estimated from the EBSD data.

**Figure 3:**
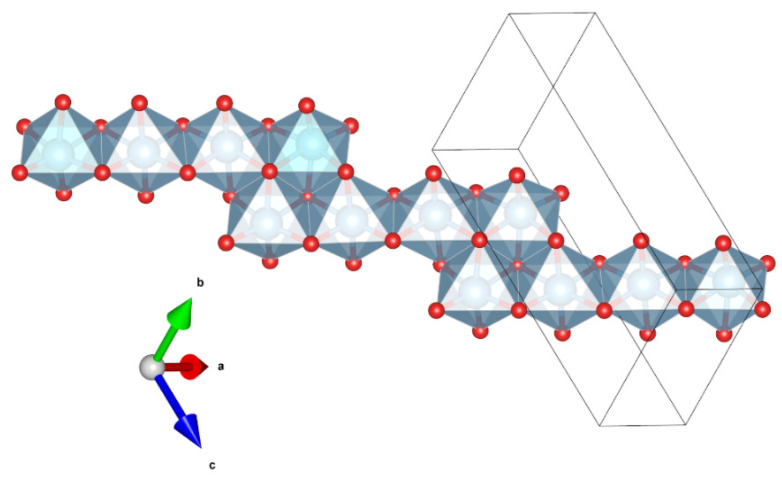
Detail of the structure of magnéliite (using the atom coordinates form Marezio and Dernier, 1971) projected onto (−556), showing the chains of four-member units of edge-sharing Ti-centered octahedra. Figure obtained using Vesta 3.0 [27].

Magnéliite (Ti^3+^_2_Ti^4+^_2_O_7_) is a new Ti-oxide mineral. It belongs to the so-called Magnéli phases, i.e., a series of Ti-oxides homologous with Ti_n_O_2n−1_ (with n = from 4 to 10). The first member of the series, synthetic Ti_4_O_7_, is well known (e.g., [23,24,25]). The crystal structure of magnéliite can be considered to be derived from the structure of rutile TiO_2_ by crystallographic shear of the (121)_rutile_ plane with a 1/2[0–11]_rutile_ vector every four octahedra of rutile [26]. The resulting structure has chains of edge-sharing TiO_6_ octahedra truncated every four octahedra by the crystallographic shear planes (Figure 3). At room-*T*, Ti^3+^ and Ti^4+^ are disordered among the eight symmetrically independent positions, while at *T* < 120 K, Ti^3+^ and Ti^4+^ are arranged in an ordered fashion to form a Ti^3+^-Ti^4+^ pair (bipolarons) and the material becomes a nonmagnetic insulator. Recent data by [26] show that even at room-*T* some local ordering of Ti^3+^–Ti^3+^ and Ti^4+^–Ti^4+^ pairs exists. 

### 3.2. Ziroite

**Figure 4:**
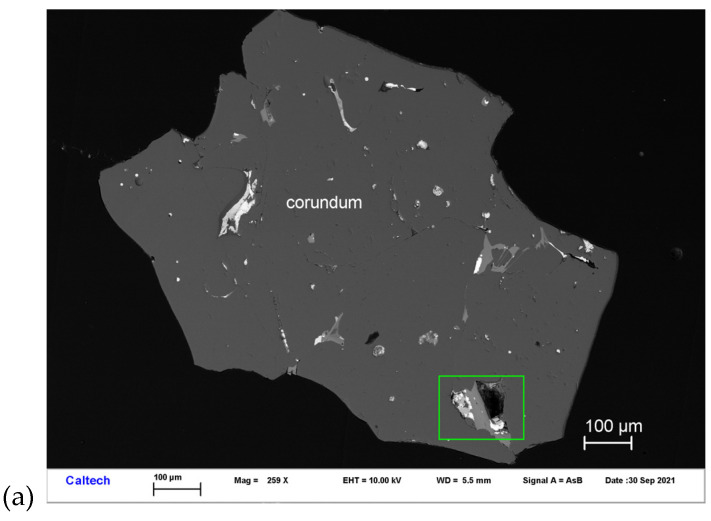

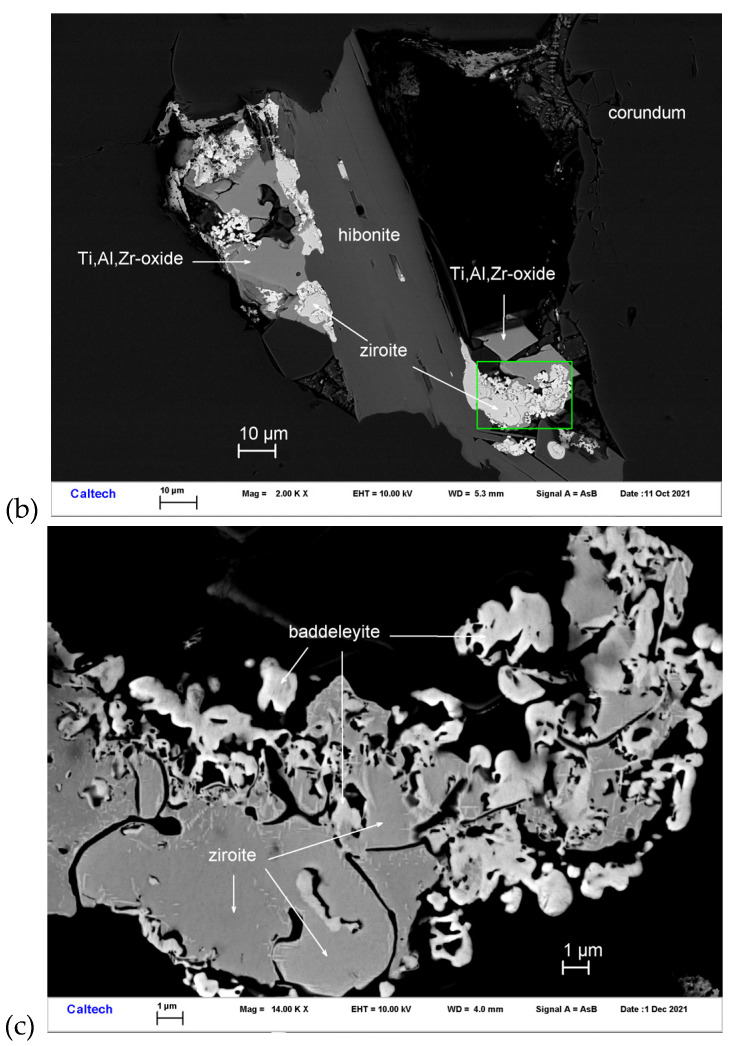
BSE images showing ziroite (ZrO_2_) in corundum Grain 479-1a. The rectangular area in (**a**) is enlarged in (**b**). The rectangular area in (**b**) is enlarged in (**c**).

Ziroite occurs with baddeleyite, Ce-rich hibonite, and Ti,Al,Zr-oxide in inclusions in corundum Grain 479-1a (Figure 4). Other inclusions in this corundum grain are MgAl-spinel, fluorbritholite-(Ce), osbornite, and hapkeite (Fe_2_Si). It occurs as irregular crystals ~1–4 μm in size. It is transparent and shows a brownish black tint. The Gladstone–Dale relationship gives n = 2.342.

The chemical composition of ziroite (Table 2) gives rise to an empirical formula (based on 2 O *pfu*) of (Zr_0.72_Ti^4+^_0.26_Mg_0.02_Al_0.02_Hf_0.01_)_Σ1.03_O_2_. The simplified formula is (Zr,Ti)O_2_. The ideal formula is ZrO_2_.

**Figure 5:**
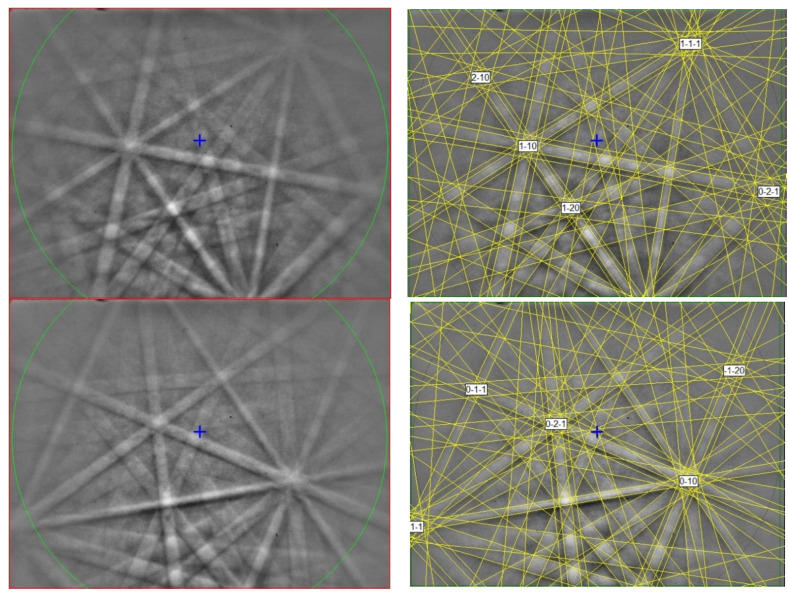
(**left**) EBSD patterns of the ziroite crystals in Figure 4, and (**right**) the patterns indexed with the *P*4_2_/nmc zirconia(HT)-type. Blue cross marks the pattern center.

The EBSD patterns can be indexed only by the tetragonal *P*4_2_/*nmc* zirconia (HT)-type and match the synthetic ZrO_2_ cell values of [28] (Figure 5), with a mean angular deviation of 0.30–0.36°, revealing the following cell parameters: *a* = 3.60(1) Å, *c* = 5.18(1) Å, *V* = 67.1(3) Å^3^, and *Z* = 2. The calculated density is 5.53 g·cm^−3^ using the empirical formula and the unit-cell volume estimated from the EBSD data.

**Figure 6:**
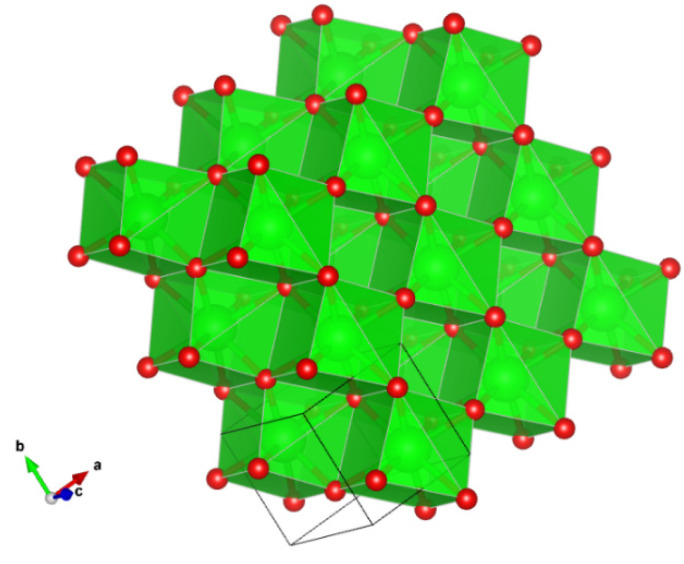
The structure of ziroite. Figure obtained using Vesta 3.0 [27].

Ziroite is a tetragonal polymorph of baddeleyite (monoclinic ZrO_2_). Synthetic ZrO_2_ with the *P*4_2_/*nmc* zirconia (HT)-type structure is well known (Figure 6) (e.g., [28,29,30]). Reported here is the first natural occurrence of tetragonal ZrO_2_. 

### 3.3. Sassite

**Figure 7:**
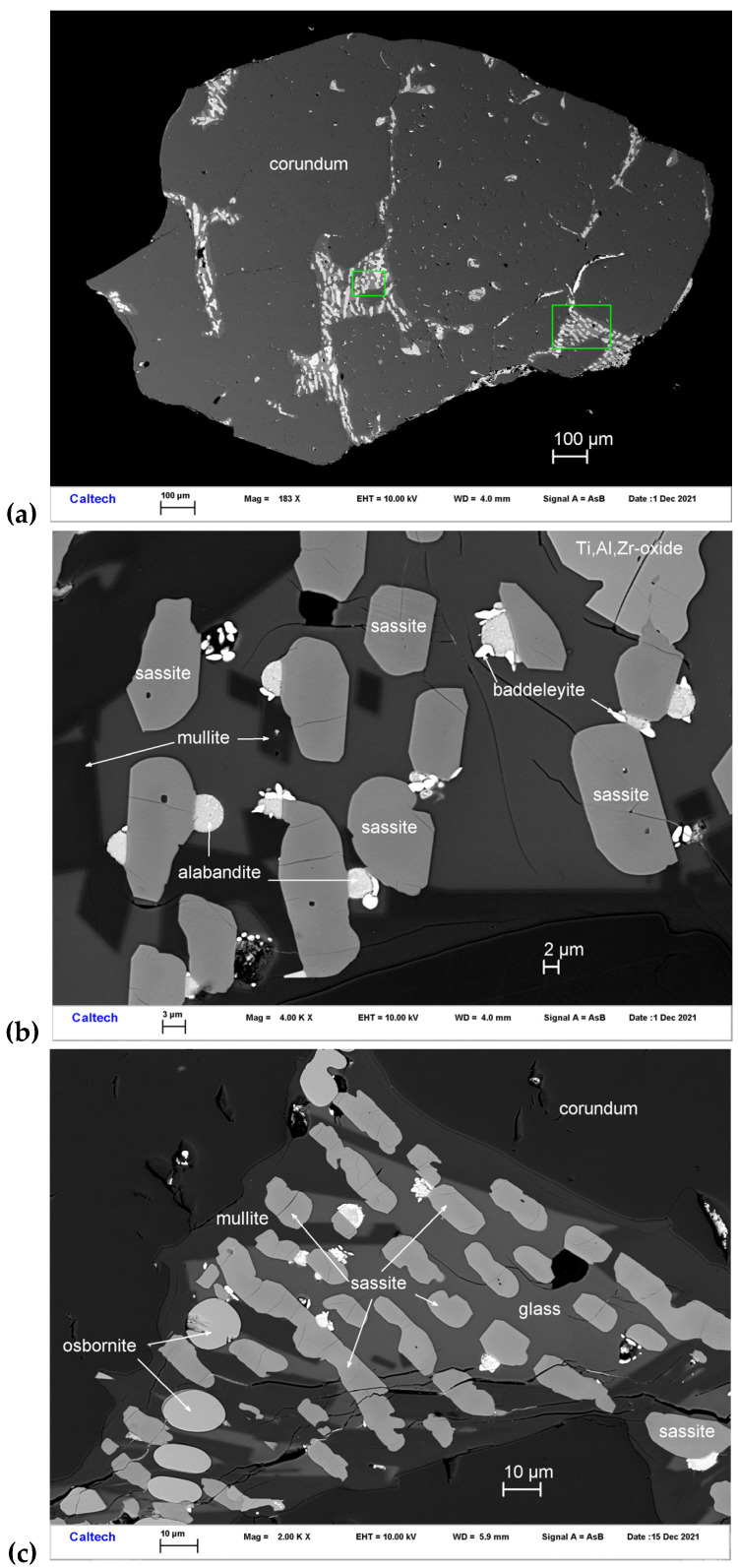
BSE images showing sassite (Ti^3+^_2_Ti^4+^O_5_) in corundum Grain 1125C1. The rectangular area in (**a**) is enlarged in (**b**). The rectangular area in (**b**) is enlarged in (**c**).

Sassite occurs with Ti,Al,Zr-oxide, mullite, osbornite, baddeleyite, alabandite, and Si-rich glass in melt pockets trapped in corundum Grain 1125C1 (Figure 7). The mineral occurs as subhedral-euhedral crystals ~4–16 μm in size. It is transparent with a brown color. The Gladstone–Dale relationship gives n = 2.16. 

Sassite (Table 3) shows an empirical formula (based on 5 O *pfu*) of (Ti^3+^_1.35_Al_0.49_Ti^4+^_0.08_Mg_0.07_)_Σ1.99_(Ti^4+^_0.93_Zr_0.06_Si_0.01_)_Σ1.00_O_5_. The simplified formula is (Ti^3+^, Al)_2_Ti^4+^O_5_. The ideal formula is Ti^3+^_2_Ti^4+^O_5_, which requires Ti_2_O_3_ 64.29, TiO_2_ 35.71, total 100 wt%.

**Figure 8:**
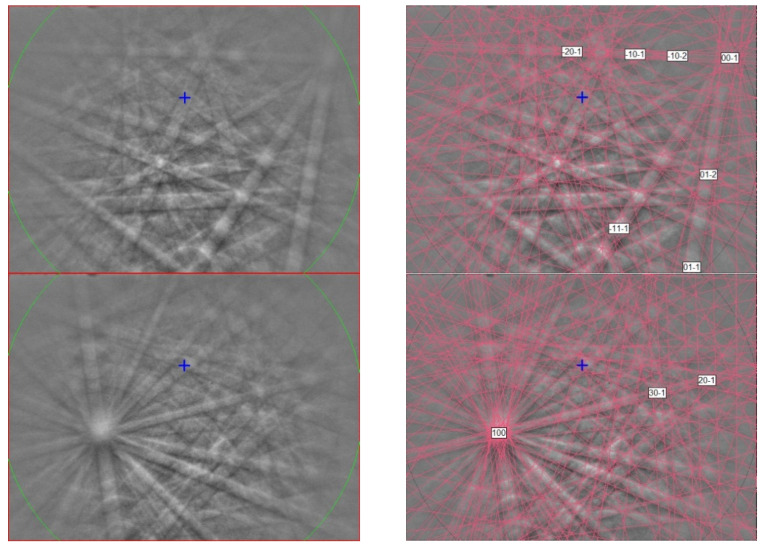
(**left**) EBSD patterns of two sassite crystals in Figure 7, and (**right**) the patterns indexed with the *Cmcm* pseudobrookite-type Ti_3_O_5_ structure. Blue cross marks the pattern center.

The EBSD patterns can be indexed only by the orthorhombic *Cmcm* pseudobrookite-type structure and match the synthetic β-Ti_3_O_5_ cell from [31] (Figure 8), with a mean angular deviation of 0.31°–0.35°, revealing the following cell parameters: *a* = 3.80(1) Å, *b* = 9.85(1) Å, *c* = 9.99(1) Å, *V* = 374(1) Å^3^, and *Z* = 4. The calculated density is 3.81 g·cm^−3^ using the empirical formula and the unit-cell volume estimated from the EBSD data.

Sassite (Ti^3+^_2_Ti^4+^O_5_) is a new member of the pseudobrookite group, joining pseudobrookite (Fe_2_TiO_5_), armalcolite [(Mg,Fe^2+^)Ti_2_O_5_], and griffinite (Al_2_TiO_5_; IMA 2021-110) [14]. Synthetic Ti_3_O_5_-pseudobrookite is well known (e.g., [31,32]). It is also known as β-Ti_3_O_5_ because several polymorphs of Ti_3_O_5_ have been described so far. α–Ti_3_O_5_ has monoclinic symmetry, *C*2/*m*, with *a* = 9.752(1), *b* = 3.802(1), *c* = 9.442(1) Å, and β = 91.55(1)° [33]. β-Ti_3_O_5_ has orthorhombic symmetry, *Cmcm*, *a* =3.798(2), *b* = 9.846(3), *c* = 9.988(4) Å [31], which corresponds to sassite. γ-Ti_3_O_5_ has the monoclinic V_3_O_5_-type structure, with *a* = 10.115, *b* = 5.074, *c* = 7.182 Å, β = 112°, and *C*2/*c* space group [34], which corresponds to the mineral kaitianite, recently described in Allende CV3 carbonaceous chondrite [35]. δ–Ti_3_O_5_ has a monoclinic structure and *P*2/*a* space group, with lattice parameters *a* = 9.9651(7), *b* = 5.0604(4), *c* = 7.2114(5) Å, and β = 109.3324(9)°, and is related to γ-Ti_3_O_5_ by decreasing temperature [36,37]. λ–Ti_3_O_5_ is monoclinic *C*2/*m* with *a* = 9.8357(11), *b* = 3.7935(2), *c* = 9.9863(7) Å, β = 90.976(6)°, and is related to α–Ti_3_O_5_ by a second-order phase transition [38].

**Figure 9:**
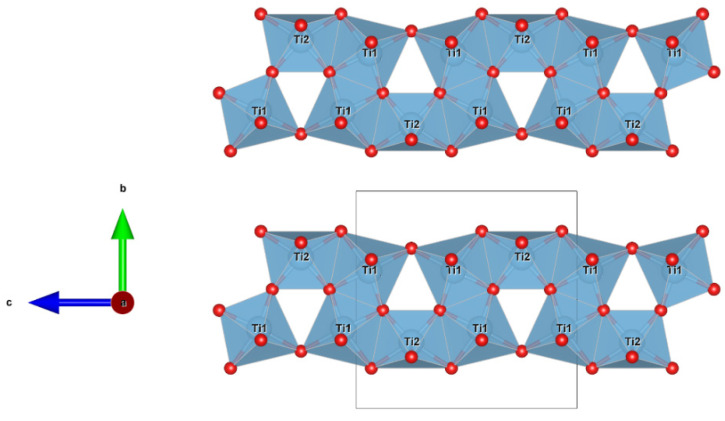
Detail of the structure of sassite (using atom coordinates form Onoda 1998) projected onto (010), showing the chains of TiO_6_ octahedra along [001] sharing edges and vertexes. Figure obtained using Vesta 3.0 [27].

Kaitianite was first discovered in association with tistarite and rutile together with other refractory phases of corundum, xifengite, mullite, osbornite, and a new Ti,Al,Zr-oxide mineral in the Allende meteorite [35]; most of these minerals are also found in the melt pockets in corundum from Mt. Carmel. Ti^3+^-rich phases are common in melt inclusions in those corundum xenocrysts, including tistarite (Ti_2_O_3_), magnéliite (Ti^3+^_2_Ti^4+^_2_O_7_), sassite (Ti^3+^_2_Ti^4+^O_5_), and grossmanite. Sc-bearing sassite was identified in the SaU 290 CH3 chondrite as an ultrarefractory phase, labeled as “anosovite,” among the first solids formed in the solar system [39]. While low-temperature α–Ti_3_O_5_ is highly ordered (three octahedrally Ti sites, with <Ti–O> = 2.015 Å, 2.033 Å, and 2.033 Å), both sassite and kaitianite show higher degrees of disorder (two crystallographically independent Ti sites, labeled Ti1 and Ti2, are octahedrally surrounded by oxygen atoms, having <Ti1–O> = 2.0271 Å and <Ti2–O> = 2.0385 Å in sassite [31] and <Ti1–O> = 2.0334 Å and <Ti2–O> = 2.0331 Å in kaitianite [34]). In sassite, the TiO_6_ octahedra are linked by sharing edges and corners, building up a characteristic row extending along the *c* axis, which is joined to an adjacent row along [100] by sharing edges (Figure 9).

### 3.4. Mizraite-(Ce)

**Figure 10:**
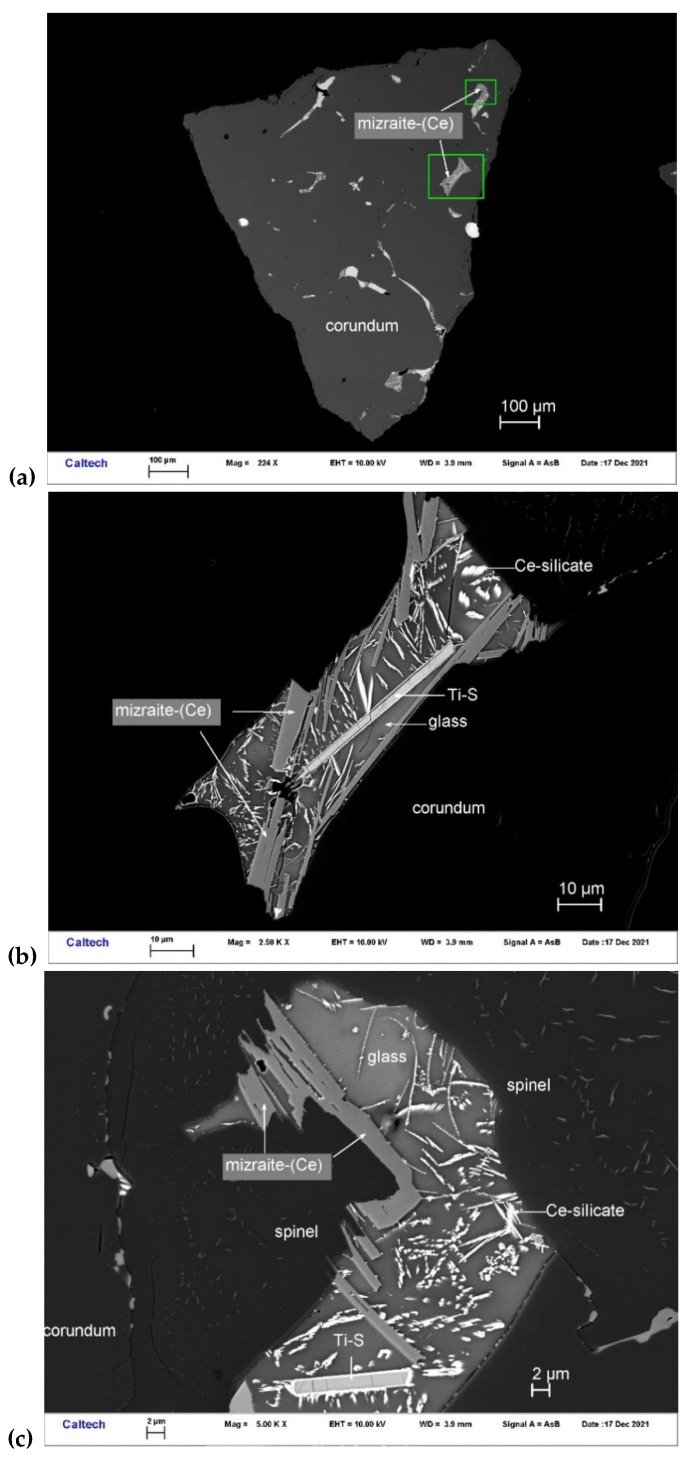
BSE images showing mizraite-(Ce) in corundum Grain 198-8. The rectangular areas in (**a**) are enlarged in (**b**,**c**).

Mizraite-(Ce) occurs with Ce-silicate and Ti-sulfide in melt pockets between corundum and spinel within Grain 198-8 (Figure 10). Other inclusions in this corundum grain contain Ti,Al,Zr-oxide, ziroite, baddeleyite, thorianite, osbornite, zangboite (TiFeSi_2_), wenjiite (Ti_5_Si_3_), and a [(Mn,Fe,Ti,V,Cr)_4_Ti_2_]Si_5_ alloy. The mineral occurs as euhedral crystals < 1–14 μm in size. It is transparent with a light bluish-green color. The Gladstone–Dale relationship gives n = 1.828.

Mizraite-(Ce) (Table 4) exhibits an empirical formula (based on 19 O *pfu*) of (Ce_0.76_Ca_0.10_La_0.07_Nd_0.01_)_Σ0.94_(Al_10.43_Mg_0.84_Ti^3+^_0.60_Si_0.09_Zr_0.04_)_Σ12.00_O_19_. The simplified formula is (Ce,Ca,La)(Al,Mg,Ti^3+^)_12_O_19_. The ideal formula is Ce(Al_11_Mg)O_19_, which requires Ce_2_O_3_ 21.45, Al_2_O_3_ 73.28, MgO 5.27, total 100 wt%.

**Figure 11:**
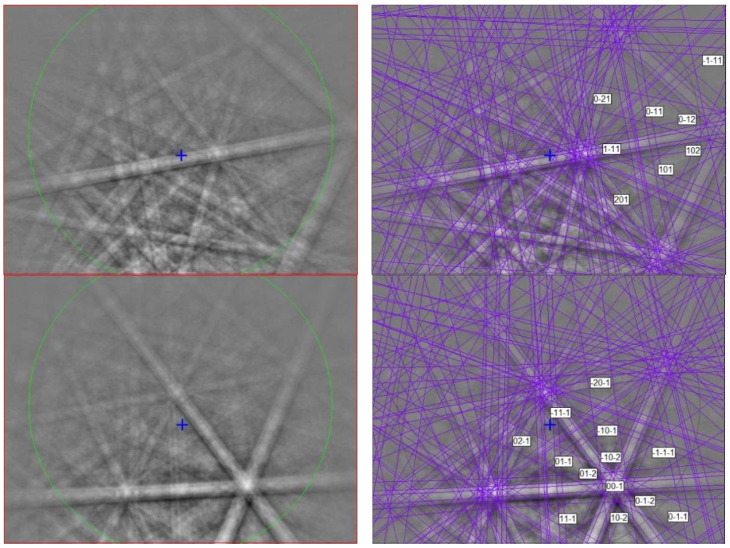
(**left**) EBSD patterns of two mizraite-(Ce) crystals in Figure 10, and (**right**) the patterns indexed with the *P*6_3_/*mmc* hibonite structure. Blue cross marks the pattern center.

The EBSD patterns can be indexed only by the hexagonal *P*6_3_/*mmc* magnetoplumbite structure and match the Ce-bearing hibonite cell of [40] (Figure 11), with a mean angular deviation of 0.32°–0.37°, revealing the following cell parameters: *a* = 5.61(1) Å, *c* = 22.29(1) Å, *V* = 608(2) Å^3^, and *Z* = 2. The calculated density is 4.16 g·cm^−3^ using the empirical formula and the unit-cell volume estimated from the EBSD data.

**Figure 12:**
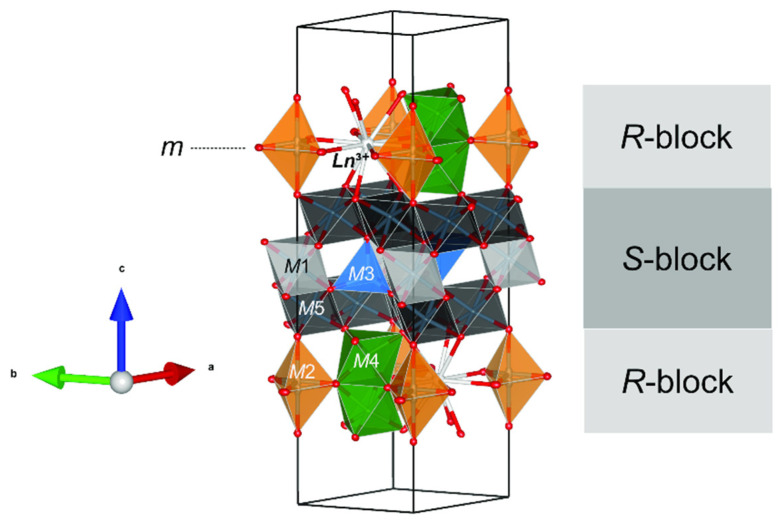
Detail of magnetoplumbite-type structure of mizraite-(Ce), showing the interlayering of *S*- and *R*-blocks. *Ln*^3+^ cations are located in the *R*-blocks along with the *M*2 and *M*4 sites. Figure obtained using Vesta 3.0 [27].

Mizraite-(Ce) is the Ce-analog of hibonite, and is a new member of the magnetoplumbite group (*A*[*B*_12_]O_19_; [16]); it is the first member presenting the heterovalent substitution *A*^2+^ + *B*^3+^ → *A*^3+^ + *B*^2+^ (Ca^2+^ + Al^3+^ → *REE*^3+^ + Mg^2+^) as the dominant species-defining exchange. Whenever another magnetoplumbite REE-dominant mineral is described, it would represent a new subgroup along with the magnetoplumbite (*A* = Pb), hawthorneite (*A* = Ba), and hibonite (*A* = Ca) subgroups. Hibonite has a general formula of (Ca,Ce)(Al,Ti,Mg)_12_O_19_ and an ideal formula of CaAl_12_O_19_. Synthetic Ce(Al_11_Mg)O_19_ is not reported, whereas La(Al_11_Mg)O_19_, La(Al_11_Mn)O_19_, and La(Al_11_Ni)O_19_ with the hibonite structure have been synthesized [41,42,43]. Terrestrial hibonite often contains minor Ce and other *REE*s and has a general formula of (Ca,Ce)(Al,Ti,Mg)_12_O_19_ [40,44]. Reported here is the first natural occurrence of Ce(Al_11_Mg)O_19_, although zoned “hibonite” grains with *REE*-rich cores (Σ*REE* > 0.6 atoms per formula unit) have been described by [45] where kalsilite, leucite, and hibonite occur together with spinel, corundum, sphene, perovskite, Ti-phlogopite, and K-feldspar in a granulite-facies gneiss in the Punalur district in Kerala, southern India. The structure of mizraite-(Ce) has the topology of the magnetoplumbite group minerals with *Ln*^3+^(Al_11_*M*^2+^)O_19_ stoichiometry and is made of two structural layers: the hexagonal close-packed *R*-block, containing the *Ln*^3+^ site, the trigonal bipyramidal *M*2 site, and the octahedral face-sharing *M*4 site; and the cubic close-packed *S*-block, containing layers of *M*5 octahedra interspaced by the *M*3 tetrahedra and the *M*1 octahedra (Figure 12). The spinel blocks contain most of the Al^3+^ in the *M*1 and *M*5 sites, and *M*^2+^ cations are distributed among the octahedral and tetrahedral sites. The *Ln*^3+^ and remaining Al^3+^ cations are localized in mirror planes, whereas *M*4 octahedra containing high-charge small cations lie on both sides of the mirror plane (Figure 12). The separation between the two *Ln*^3+^ sites of the same mirror plane is equal to the *a* unit cell parameter (ca. 5.6 Å), whereas between two different mirror planes it is approximately 11 Å.

### 3.5. Yeite

**Figure 13:**
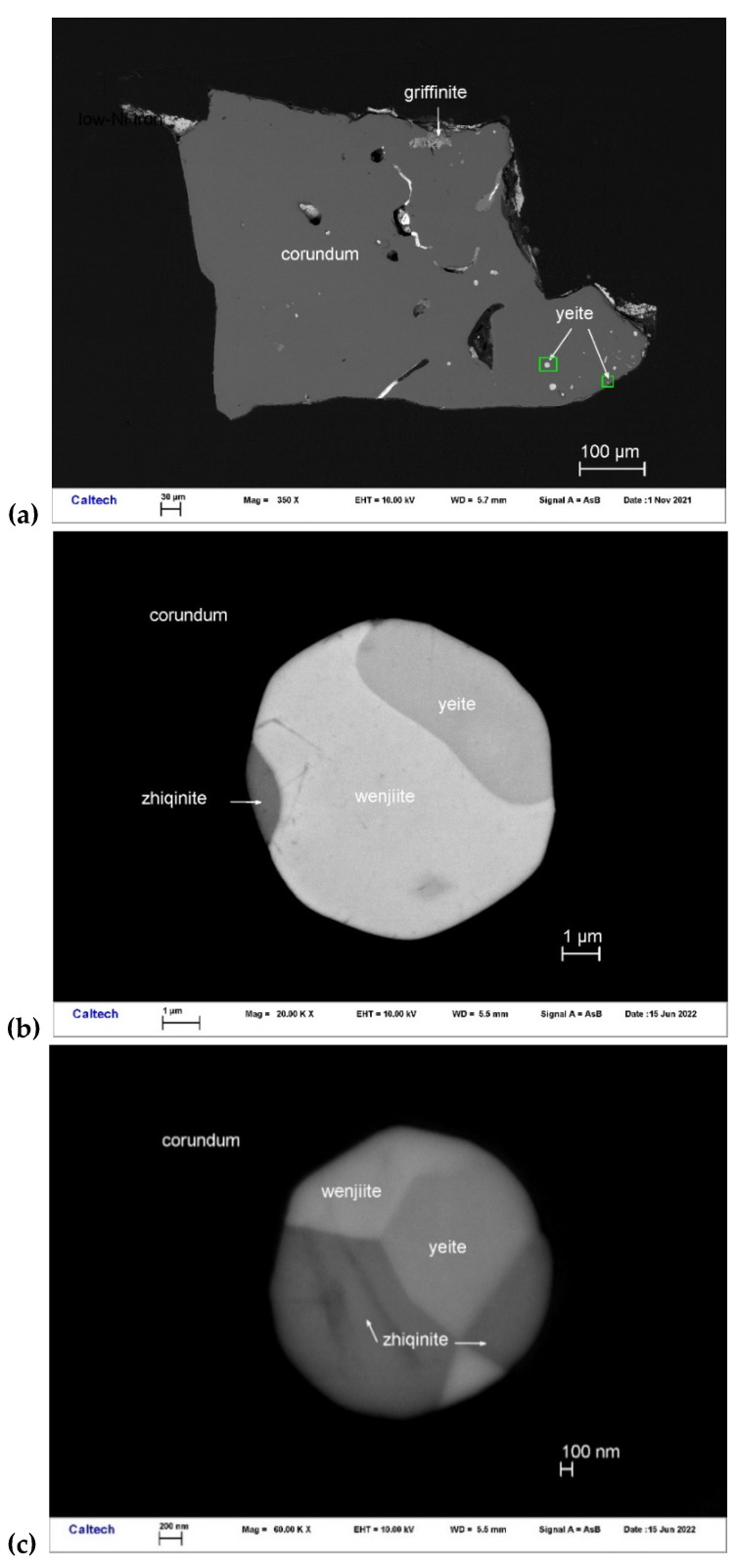
BSE images showing yeite (TiSi) with wenjiite (Ti_5_Si_3_) and zhiqinite (TiSi_2_) in corundum Grain 198c. The rectangular areas in (**a**) are enlarged in (**b**,**c**).

Yeite occurs with wenjiite (Ti_5_Si_3_) and zhiqinite (TiSi_2_) in Ti-Si alloy inclusions in corundum Grain 198c (Figure 13). Other inclusions in this corundum grain contain type griffinite (Al_2_TiO_5_) [14], rutile, baddeleyite, hibonite, osbornite, khamrabaevite, Ti,Al,Zr-oxide, zirconolite, and jingsuiite. Yeite occurs as irregular-subhedral crystals 1.2–3.5 μm in size. It is opaque and shows a black color.

The chemical composition of yeite (Table 5) gives rise to an empirical formula (based on 2 atoms *pfu*) of (Ti_0.995_Mn_0.003_V_0.001_Cr_0.001_)(Si_0.996_P_0.004_). The simplified formula is TiSi. The ideal formula is TiSi, which requires Ti 63.04, Si 36.96, total 100 wt%. Associated wenjiite has an empirical formula (Ti_3.70_Mn_0.43_Cr_0.07_V_0.02_)Si_3.68_. Zhiqinite has an empirical formula Ti_0.99_Si_2.01_.

**Figure 14:**
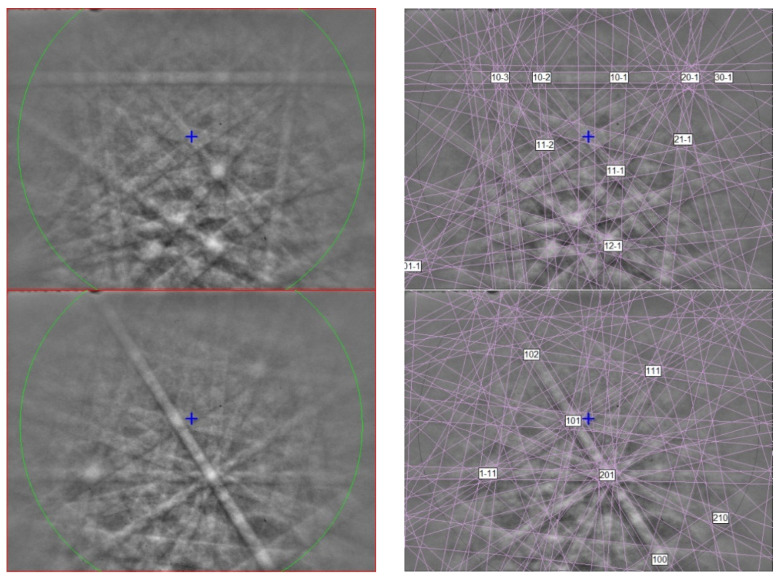
(**left**) EBSD patterns of yeite in Figure 13 at different orientations, and (**right**) the patterns indexed with the *Pnma* TiSi structure. Blue cross marks the pattern center.

The EBSD patterns of yeite can be indexed only by the orthorhombic *Pnma* FeB-type structure and match the synthetic TiSi cells of [46,47] (Figure 14), with a mean angular deviation of 0.23°–0.32°, revealing the following cell parameters: *a* = 6.55(1) Å, *b* = 3.64(1) Å, *c* = 4.99(1) Å, *V* = 119.0(4) Å^3^, and *Z* = 4. The calculated density is 4.24 g·cm^−3^ using the empirical formula and the unit-cell volume estimated from the EBSD data.

**Figure 15:**
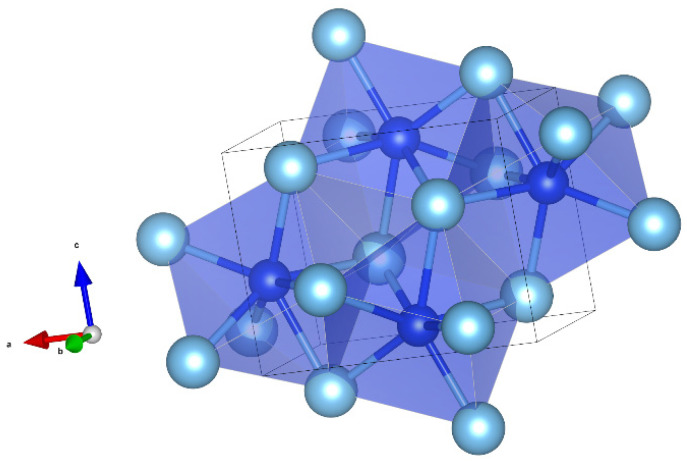
The structure of yeite (using the atom coordinates published by [46]). SiTi_7_ capped triangular prims in blue. The polyhedra share edges. Figure obtained using Vesta 3.0 [27].

In the structure of yeite, each Si atom is coordinated with seven Ti atoms, forming SiTi_7_ polyhedra (capped trigonal prism, <Si-Ti> = 2.638 Å, distortion index = 0.01259) that share edges, building up a three-dimensional framework (Figure 15). The structure can be also described as TiSi_7_ polyhedra (<Si-Ti> = 2.636 Å, distortion index = 0.01245), with an odder shape, sharing edges.

Yeite is natural TiSi with the *Pnma* FeB-type structure. Synthetic TiSi with the *Pnma* FeB-type structure is well known [46,47]. To our knowledge, yeite is not related to other minerals. Other Ti-Si minerals include zhiqinite with an orthorhombic *Fddd* TiSi_2_-type structure [48], kangjinlaite (Ti_11_Si_10_) with a tetragonal *I*4/*mmm* Ho_11_Ge_10_-type structure [49], and a special wenjiite (Ti_5_Si_3_) with a hexagonal *P*6_3_/*mcm* Mn_5_Si_3_-type structure identified in this study.

## 4. Discussion

The oxide minerals described here are high-temperature phases. They crystallized from melts that were trapped in intracrystalline and interstitial voids in aggregates of corundum crystals [2]. The whole suite of corundum xenoliths is characterized by oxygen fugacity (*f*O_2_) below the levels normally encountered in Earth’s upper mantle or crust (IW to IW-9; [50]). We recognize three broad paragenetic types.

*Crn-A*: these are hopper to skeletal crystals showing strong zoning in Ti due to the uptake of Ti^3+^ during rapid crystal growth [51]. The composition of the trapped melts is Ca-Mg-Al silicates showing high contents of S as well as incompatible elements. Phase assemblages reflect low *f*O_2_, with all Ti as Ti^3+^ (e.g., tistarite).

*Crn-B*: these are large homogeneous (unzoned) corundum crystals, which typically show Ti contents > 1 wt%. In these crystals, interstitial pockets contain small amounts of glass, which are typically high in *REE*, Zr, and other incompatible elements. In phenocrysts, Ti is present as both Ti^3+^ and Ti^4+^.

*Crn-C*: these are texturally similar to *Crn-B*; however, the Ti contents in corundum are typically low (<0.5 wt% Ti). Rare glasses are rich in *LREE* and Ba. The presence of more Ti^4+^ phases (rutile, griffinite) suggests higher mean *f*O_2_ than in *Crn-A* and *Crn-B*. Hibonite occurs in all three parageneses; in *Crn-A* and *Crn-B*, it contains high levels of Ti^3+^, whereas in *Crn-C,* the Ti^3+^ contents are very low.

**Figure 16:**
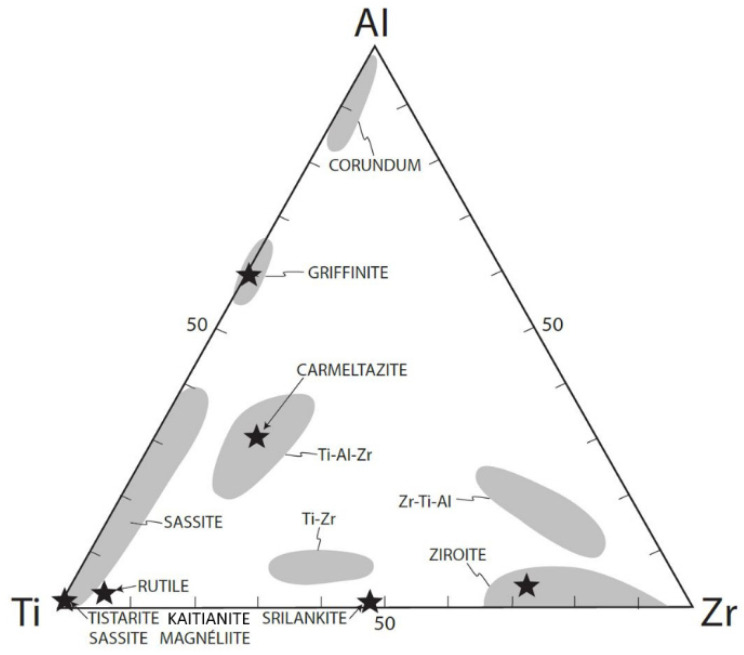
Ti-Al-Zr triplot showing phases from melt inclusions in corundum xenocrysts from the Mt. Carmel area, from [52].

Magnéliite, sassite, and ziroite are members of a large population of Ti-Al-Zr phases, which include carmeltazite, griffinite, tistarite, rutile, “Allende-like” Ti-Zr-Al oxide [35], kaitianite [52], and many as yet undescribed minerals (Figure 16). Part of this variety is due to the presence of Ti as both Ti^3+^ and Ti^4+^, reflecting the differences in *f*O_2_ among the three parageneses. Individual phases may show large ranges in solid solution, reflecting substitutions of trivalent (Ti^3+^, Al) and quadrivalent (Ti^4+^, Zr) ions.

Magnéliite shows some solid solution of both ZrO_2_ and Al_2_O_3_ (Table 1); it has crystallized from a glass, residual after the crystallization of large hibonite crystals. The type magnéliite is associated with alabandite, which suggests that both crystallized during the ascent of the xenoliths as decreasing pressure led to lower solubility of sulfur in the melt. This assemblage and the low Ti in corundum (0.4 wt% Ti) are characteristic of paragenesis *Crn-C*.

Sassite is clearly a liquidus phase (Figure 4) together with a Ti-Al-Zr oxide and corundum; the reconstructed melt in these interstitial pockets is low in Si and Ca and very high in Ti, while the residual melt is Al, Si-rich. Sassite shows a very wide range of solid solution toward griffinite (Al_2_TiO_5_ [14]; Figure 16). The presence of alabandite and baddeleyite suggests quench crystallization during ascent of the xenolith, which is consistent with the quench crystallization of mullite at low P. This is a typical *Crn-B* paragenesis.

Ziroite can have a significant solid solution of TiO_2_ (Table 3; Figure 16). The ability of ziroite to take up Ti can explain the coexistence of ziroite and baddeleyite (Figure 7), as the latter does not appear to take up much Ti. Like sassite, the type ziroite has crystallized from a Ca-Mg-Al-silicate glass, residual after the crystallization of hibonite and a Ti-Al-Zr oxide, in a typical *Crn-B* paragenesis.

Mizraite-(Ce) is also clearly a liquidus phase, crystallizing from a residual melt high in *LREE* and S. Its occurrence as interstitial to large exsolved spinel grains suggests that it belongs to paragenesis *Crn-B*, although the low Ti content of the adjacent corundum (0.4 wt%) is more characteristic of *Crn-C*.

The study of mixed-valence phases in paragenesis *Crn-B*, and possibly in *Crn-C*, provides new information on the interpretation of the origins of the Mt. Carmel corundum-aggregate xenoliths. While the different parageneses share many common features, it has proven difficult to establish common lines of descent between them.

The alloy phases, including yeite as described here, appear as inclusions in aggregates of corundum crystals; they represent trapped melts, melts + crystals, and subsolidus assemblages that formed from the melts on cooling, both prior to eruption and during quenching upon eruption of the host basalts [6]. The immiscible separation of these melts from the coexisting silicate melt under highly reducing conditions allowed the crystallization of Fe-free phases from the silicate melt(s). The chemistry and evolution of these melts through multiple stages of immiscibility have been described in [6]; yeite adds more detail to this picture.

**Figure 17:**
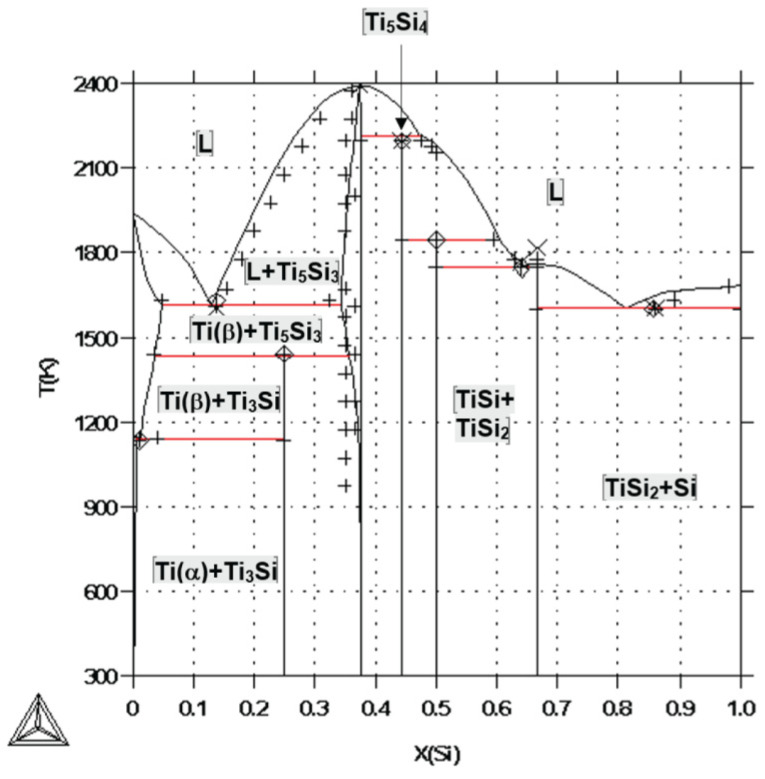
Calculated Ti-Si phase diagram from [53].

Yeite occurs in spheroidal balls (Figure 13) interpreted as immiscible melts coexisting with the silicate melt from which the enclosing corundum was crystallizing. The smooth, straight, or irregular boundaries between yeite, wenjiite, and zhiqinite suggest that the original melt may have decomposed into mutually immiscible melts or crystallized into the three coexisting phases. However, examination of the phase diagram for the Ti-Si Si binary [53] suggests that the situation was more complex (Figure 17).

**Figure 18:**
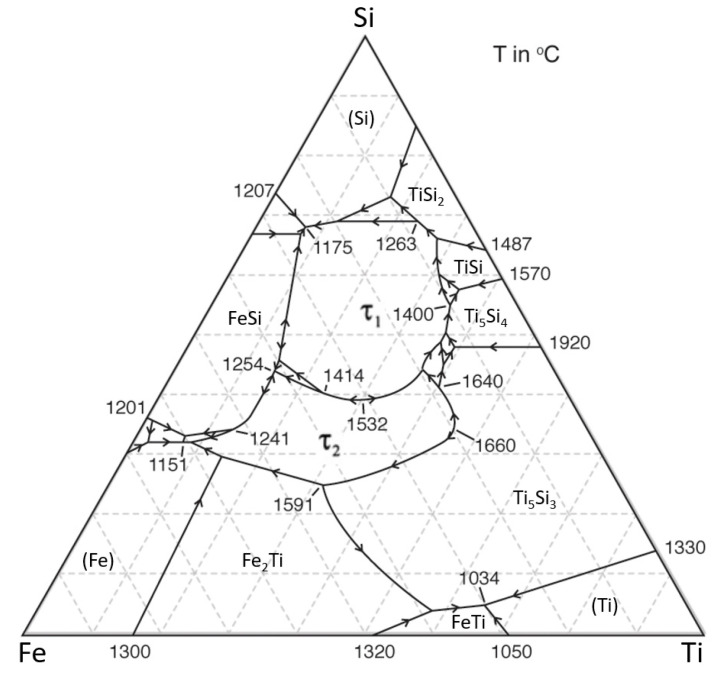
Liquidus projection for Fe-Si-Ti, modified from [54].

This binary is separated into two subsystems by a thermal divide at Ti_3_Si_2_; the assemblage TiSi+TiSi_2_ appears (crystallizes) at a eutectic point (1743 K) on the Si side of the divide, while wenjiite crystallizes from melts on the Ti side of the divide from 2400 K to a eutectic (L → Ti + Ti_5_Si_3_) at 1613 K. There is no point at which TiSi coexists with Ti_5_Si_3_. However, the average reconstructed composition of the melts in Figure 13 lies near several cotectics (1773–1673 K) in the Fe-Ti-Si ternary system (Figure 18) [54], making it probable that three phases may have crystallized from the melt over a very short *T* range in the high-temperature part of this ternary system. As noted by [6], the temperatures in the natural system beneath Mt. Carmel probably were lower than those in the synthetic systems due to the coexistence of a fluid phase rich in H_2_, which can lower temperatures in metallic systems by up to several hundred degrees [55].

These alloy minerals thus illustrate the wide range of immiscible-melt compositions and crystallization conditions captured in the xenoliths from Mt. Carmel and give some new insights into processes in this highly reduced magmatic system. This highly reduced corundum-related assemblage is not simply a one-locality oddity; very similar associations have been reported from the Luobusa ophiolite in SE Tibet [48,49] and from many other localities in intraplate and subduction-zone tectonic settings [2,6]. These occurrences imply a significant role for mantle-derived CH_4_+H_2_ fluids in magmatic processes.

## 5. Conclusions

Reported here is the discovery of five new minerals, magnéliite (Ti^3+^_2_Ti^4+^_2_O_7_), ziroite (ZrO_2_), sassite (Ti^3+^_2_Ti^4+^O_5_), mizraite-(Ce) (Ce(Al_11_Mg)O_19_), and yeite (TiSi), in melt inclusions in corundum xenocrysts from the Mt. Carmel area, Israel. The description of their chemical composition and the crystal structures of the synthetic analogues that match the EBSD data is provided. Many physical properties cannot be obtained because of the extremely reduced dimensions of the grains (nano scale), but the data are sufficient to support their correct identification. These minerals are high-temperature oxide or alloy phases formed under extremely reduced conditions in the upper mantle, and provide new, important insights into the natural origin of super-reduced mineral assemblages.

## Figures and Tables

**Table 1:** EPMA analytical results (in wt%, n = 7) for magnéliite.

Constituent	Mean	Range	SD (σ)	Probe Standard
TiO_2_ *	53.54	53.28–53.93	0.17	TiO_2_
Ti_2_O_3_ *	38.63	38.44–38.84	0.12	TiO_2_
ZrO_2_	3.02	2.86–3.20	0.11	zircon
Al_2_O_3_	2.20	2.03–2.26	0.08	anorthite
MgO	1.36	1.26–1.51	0.09	forsterite
CaO	0.19	0.17–0.20	0.01	anorthite
Sc_2_O_3_	0.22	0.20–0.23	0.01	ScPO_4_
Total	99.16			

* Total titanium has been partitioned between Ti^3+^ and Ti^4+^ for charge balance to make ideal stoichiometry.

**Table 2:** EPMA analytical results (in wt%, n = 8) for ziroite.

Constituent	Mean	Range	SD (σ)	Probe Standard
ZrO_2_	78.52	76.06–79.92	1.18	zircon
TiO_2_	18.21	16.68–20.47	1.18	TiO_2_
HfO_2_	1.43	1.35–1.54	0.08	Hf metal
MgO	0.70	0.62–0.75	0.05	forsterite
Al_2_O_3_	0.87	0.81–0.95	0.05	anorthite
Total	99.73			

**Table 3:** EPMA analytical results (in wt%, n = 13) for sassite.

Constituent	Mean	Range	SD (σ)	Probe Standard
Ti_2_O_3_ *	45.32	44.81–45.94	0.30	TiO_2_
TiO_2_ *	37.32	36.90–37.83	0.24	TiO_2_
Al_2_O_3_	11.73	10.22–13.02	0.83	anorthite
ZrO_2_	3.70	3.05–5.04	0.68	zircon
MgO	1.29	0.97–1.66	0.24	forsterite
SiO_2_	0.24	0.15–0.46	0.08	anorthite
MnO	0.15	0.10–0.20	0.04	Mn_2_SiO_4_
CaO	0.07	0.05–0.12	0.02	ScPO_4_
Total	99.82			

* Total titanium has been partitioned between Ti^3+^ and Ti^4+^ for charge balance to achieve ideal stoichiometry.

**Table 4:** EPMA analytical results (in wt%, n = 8) for mizraite-(Ce).

Constituent	Mean	Range	SD (σ)	Probe Standard
Al_2_O_3_	69.75	69.41–70.07	0.23	Al_2_O_3_
Ce_2_O_3_	16.30	16.13–16.50	0.14	CePO_4_
* Ti_2_O_3_	5.67	5.64–5.75	0.04	TiO_2_
MgO	4.45	4.37–4.51	0.04	forsterite
La_2_O_3_	1.47	1.40–1.56	0.06	LaPO_4_
SiO_2_	0.72	0.69–0.79	0.03	anorthite
CaO	0.71	0.69–0.73	0.02	anorthite
ZrO_2_	0.67	0.43–0.81	0.12	zircon
Nd_2_O_3_	0.29	0.25–0.35	0.04	NdPO_4_
Total	100.02			

* Titanium has been assigned to be Ti^3+^ for charge balance to achieve best stoichiometry.

**Table 5:** EPMA analytical results (in wt%, n = 7) for yeite.

Constituent	Mean	Range	SD (σ)	Probe Standard
Ti	62.34	62.11–62.71	0.21	Ti metal
Si	36.59	36.47–36.78	0.10	Si metal
P	0.18	0.13–0.25	0.05	GaP
Mn	0.18	0.14–0.31	0.06	Mn_2_SiO_4_
Cr	0.08	0.00–0.13	0.04	Cr metal
V	0.06	0.00–0.17	0.06	V metal
Fe	0.00	0.00	0.00	Fe metal
Total	99.43			

## Data Availability

Data are contained within the article.
